# Improving mental health policy in the case of schizophrenia in Thailand: evidence-based information for efficient solutions

**DOI:** 10.1186/1471-2458-12-S2-A32

**Published:** 2012-11-27

**Authors:** Pudtan Phanthunane, Theo Vos, Harvey Whiteford, Melanie Bertram

**Affiliations:** 1 Faculty of Business, Economics and Communications, Naresuan University, Phitsanulok, Thailand; 2 Ministry of Public Health, Nonthaburi, Thailand; 3 School of Population Health, the University of Queensland, Herston, Queensland, Australia; 4 Queensland Centre for Mental Health Research, The Park, Wacol, Queensland, Australia

## Background

This study aimed to provide policy makers with important information with regard to care of schizophrenia patients in Thailand. Specifically, it aimed to (a) estimate the economic burden of schizophrenia; (b) identify prioritized and effective schizophrenia treatments; and c) explore patients’ and clinicians’ perspective of schizophrenia outcomes.

## Materials and methods

This study was conducted at a national level, using the government, patients and family’s perspectives. Both primary and secondary data were used. A cross-sectional survey was conducted in the year 2008. Data were also obtained from both national and international literature and databases. The concepts of Disability Adjusted Life Year, Cost of Illness estimation and Generalized Cost-Effectiveness Analysis were adopted to estimate burden of schizophrenia and cost-effective interventions.

## Results

The prevalence of schizophrenia at ages 15-59 in the Thai population was 8.8 per 1,000 (95% CI: 7.2, 10.6) with a male to female ratio of 1.1. The annual cost of schizophrenia was estimated to be THB 87,000 per person (95% CI: 83,000, 92,000) with indirect costs (73%) as the main cost contributed. The most cost-effective treatment of schizophrenia was generic risperidone as first line treatment (dominant intervention), in a combination with family interventions for all patients. Clozapine would be prescribed only for severe patients. The results from the survey also suggested a modest association between patient-rated and clinician-rated outcomes. The patients rated themselves using the EuroQol-5D+ instrument, while the disease severity of each patient was rated by a psychotic nurse using the Brief Psychiatric Rating Scale-Expanded.

## Conclusion

Schizophrenia is an expensive disorder in Thailand. By providing more cost-effective interventions with a combination of generic risperidone, family interventions and clozapine would achieve a win-win solution for both the government and the patients/families. Policy makers and clinicians need to consider real needs of patients in future planning of schizophrenia management.

